# Comparison of the effects of empagliflozin and sotagliflozin on a zebrafish model of diabetic heart failure with reduced ejection fraction

**DOI:** 10.1038/s12276-023-01002-3

**Published:** 2023-06-01

**Authors:** Inho Kim, Hyun-Jai Cho, Soo Lim, Seung Hyeok Seok, Hae-Young Lee

**Affiliations:** 1 Department of Internal Medicine, Seoul National University Hospital, Seoul, Korea; 2 Department of Microbiology and Immunology, Seoul National University College of Medicine, Seoul, Korea; 3 Department of Internal Medicine, Seoul National University College of Medicine, Seoul, Korea; 4 Department of Internal Medicine, Seoul National University Bundang Hospital, Seongnam, Korea; 5 Department of Biomedical Sciences, Seoul National University College of Medicine, Seoul, Korea; 6 Cancer Research Institute, Seoul National University, Seoul, Korea

**Keywords:** Heart failure, Homeostasis

## Abstract

The sodium-glucose cotransporter 2 (SGLT2) inhibitor empagliflozin (EMPA) and dual SGLT1/2 inhibitor sotagliflozin (SOTA) are emerging as heart failure (HF) medications in addition to having glucose-lowering effects in diabetes mellitus (DM). However, the precise mechanism underlying this cardioprotective effect has not yet been elucidated. Here, we evaluated the effects of EMPA and SOTA in a zebrafish model of DM combined with HF with reduced ejection fraction (DM-HFrEF). To compare the effects of the two drugs, survival, locomotion, and myocardial contractile function were evaluated. The structural binding and modulating effects of the two medications on sodium-hydrogen exchanger 1 (NHE1) were evaluated in silico and in vitro. DM-HFrEF zebrafish showed impaired cardiac contractility and decreased locomotion and survival, all of which were improved by 0.2–5 μM EMPA or SOTA treatment. However, the 25 μM SOTA treatment group had worse survival rates and less locomotion preservation than the EMPA treatment group at the same concentration, and pericardial edema and an uninflated swim bladder were observed. SOTA, EMPA and cariporide (CARI) showed similar structural binding affinities to NHE1 in a molecular docking analysis and drug response affinity target stability assay. In addition, EMPA, SOTA, and CARI effectively reduced intracellular Na^+^ and Ca^2+^ changes through the inhibition of NHE1 activity. These findings suggest that both EMPA and SOTA exert cardioprotective effects in the DM-HFrEF zebrafish model by inhibiting NHE1 activity. In addition, despite the similar cardioprotective effects of the two drugs, SOTA may be less effective than EMPA at high concentrations.

## Diabetes: Glucose-lowering drugs protect against heart failure

Two existing drugs used to lower glucose in patients with diabetes also reduce the chances of heart failure by blocking the excessive activity of a protein involved in transport of molecules across membranes. The drugs empagliflozin and sotagliflozin, which inhibit sodium-glucose transporters in diabetes, also help protect against heart failure, but the mechanisms are unclear. Hae-Young Lee and Seung Hyeok Seok at Seoul National University, South Korea, and co-workers examined the cardio-protective effects of the drugs on zebrafish models of diabetic heart failure.They focused on sodium-hydrogen exchanger 1 (NHE1), which regulates sodium and hydrogen levels to maintain pH balance, and is increased in the heart ventricle tissue of patients with heart failure. The team found that both drugs blocked NHE1 activity, while also targeting sodium-glucose transporters. Empagliflozin was particularly effective at protecting the heart in this way.

## Introduction

The prevalence of diabetes mellitus (DM) is increasing rapidly worldwide. The number of people with DM worldwide is over 425 million and is expected to reach 700 million by 2045^1^. DM is strongly associated with cardiovascular disease (CVD). Among CV complications, heart failure (HF) increases significantly in DM patients compared with non-DM patients^2–4^. Worse still, DM is associated with an increased risk of overall mortality in HF^5^. Although DM is a crucial risk factor for HF, the effectiveness of glycemic control in preventing or managing HF has not yet been proven. Some blood-glucose-lowering drugs, such as insulin, thiazolidinedione (TZD), and dipeptidyl peptidase-4 (DPP-4) inhibitors, increase the risk of hospitalization for HF^6^. Therefore, the US Food and Drug Administration (FDA) requires cardiovascular outcome trials (CVOTs) for all candidate DM drugs^7^. Surprisingly, significant cardioprotective effects were demonstrated in two major trials of sodium-glucose cotransporter (SGLT) 2 inhibitors, empagliflozin (EMPA) and dapagliflozin^8,9^. In addition, sotagliflozin (SOTA), the first reported dual SGLT1/2 inhibitor, significantly reduced the risk of HF^10^.

SGLTs exist in two major isoforms: SGLT1 and SGLT2^11^. SGLT2 is strongly expressed in the renal proximal tubule and plays a crucial role in glucose reabsorption^11,12^. Inhibition of SGLT2 effectively reduces blood glucose by decreasing glucose reabsorption in the renal proximal tubule and increasing urinary glucose excretion. SGLT1 is mainly expressed in the small intestine, contributing to glucose uptake^13^. Dual SGLT1/2 inhibitors provide a practical hypoglycemic effect by reducing glucose absorption in the small intestine and glucose reabsorption from the proximal renal tubule through SGLT1 and SGLT2 inhibition, respectively^14^. Despite the significant cardioprotective effects of these inhibitors, it has not yet been determined whether SGLT2 inhibitors or dual SGLT1/2 inhibitors provide more effective cardiovascular protection.

Several recent studies have focused on the sodium-hydrogen exchanger 1 (NHE1) in the myocardium as an off-target ligand of SGLT2 inhibitors^15–17^. NHE1 is a transporter that exchanges H^+^ and Na^+^ and maintains intracellular pH homeostasis. Upregulation of NHE1 is found in the ventricular tissue of patients with HF^18^, and experimental HF models showed that selective inhibition of NHE1 improves cardiac function by suppressing fibrosis and hypertrophy^19^. However, it has not yet been confirmed whether SOTA can target NHE1 as other SGLT2 inhibitors do.

We have developed a zebrafish DM combined with HF with reduced ejection fraction (DM-HFrEF) model^20^. Zebrafish have the benefits of high fertility, cost-effectiveness, and physiological similarity to humans^21^. In particular, pancreatic β-cells and the heart are structurally and genetically similar to those of humans. Zebrafish larvae have transparent bodies; therefore, blood flow and cardiac contraction can be directly observed under a microscope. Moreover, zebrafish larvae are 3 to 5 mm in length and are suited for arraying into 96- and even 384-well plates, which are suitable for mass screening of survival after drug treatment. Here, we evaluated the effect of EMPA and SOTA in the DM-HFrEF zebrafish model and the role of NHE1 inhibition in the action of the two drugs.

## Materials and methods

### Zebrafish maintenance

Adult zebrafish (*Danio rerio*) were maintained at 28 °C on a 14:10 h light-dark cycle in an automatic circulating tank system and fed *Artemia* 2 times a day. Embryos were raised at 28 °C in egg water (60 μg/ml ocean salts, Sigma‒Aldrich, St. Louis, MO, USA), and experiments were performed on the hatched zebrafish embryos from 3 days post-fertilization (dpf) to 9.5 dpf. If the survival rate of zebrafish larvae at 24 hours post-fertilization (hpf) was less than 80%, that zebrafish larvae were not used in the experiment. We used wild-type (WT) zebrafish and a transgenic (*Tg, myl7:EGFP*) zebrafish strain expressing *enhanced green fluorescent protein* (*EGFP*) with cardiac *myosin light chain 7* (*myl7*) in the myocardium^22^. All animal experiments and husbandry procedures were approved by the Institutional Animal Care and Use Committee (IACUC) of Seoul National University (accession no. SNU-200310-1), and all experiments were conducted in accordance with the National Institutes of Health (NIH) *Guide for the Care and Use of Laboratory Animals*^23^. Zebrafish larvae were anesthetized by immersion in 0.016% tricaine solution (MS-222, Sigma‒Aldrich, St. Louis, MO, USA) for 5 min. Zebrafish larvae were euthanized by the hypothermic shock method, in which they were exposed to ice-cold water for at least 20 min, in accordance with American Veterinary Medical Association (AVMA) guidelines^24^.

### Production of the DM-HFrEF zebrafish model

The DM-HFrEF zebrafish model was generated as described in our previous study^20^. First, a DM-like condition was induced in zebrafish larvae using a combination of D-glucose (GLU, Sigma‒Aldrich, St. Louis, MO, USA) and streptozotocin (STZ, Sigma‒Aldrich, St. Louis, MO, USA). At 3 dpf, zebrafish larvae were immersed in egg water containing 40 mM GLU. D-mannitol (MAN, Sigma‒Aldrich, St. Louis, MO, USA) was used as an osmotic control for GLU. At 4 dpf, 50 μg/ml STZ was added in the dark, and the larvae were incubated for 2 h. At 5 dpf, HF was induced by treatment with terfenadine (TER, Sigma‒Aldrich, St. Louis, MO, USA), a potassium channel blocker that induces HF in zebrafish^20,25,26^. EMPA or SOTA (Cayman Chemical, Ann Arbor, MI, USA) was administered according to the respective concentrations at 5 dpf (Supplementary Table 1), and analyses were performed after more than 24 h of incubation (Supplementary Fig. 1).

### Quantitative real-time polymerase chain reaction (qRT‒PCR)

For qRT‒PCR analysis, euthanized zebrafish larvae were washed twice with phosphate-buffered saline (PBS), and total cellular RNA was extracted using QIAzol Lysis Reagent (Qiagen, Hilden, Germany). The extracted total cellular RNA was reverse transcribed using amfiRivert cDNA Synthesis Premix (GenDEPOT, Katy, TX, USA) according to the manufacturer’s instructions. Using the SYBR Green PCR kit (GenDEPOT, Katy, TX, USA) and the StepOnePlus Real-Time PCR System (Applied Biosystems, Waltham, MA, USA), qRT‒PCR was performed on the prepared cDNA. The following primers were used: *nppb* (forward: 5′-CAT GGG TGT TTT AAA GTT TCT CC-3′, reverse: 5′-CTT CAA TAT TTG CCG CCT TTA C-3′) and *18S ribosomal RNA* (*rRNA*) (forward: 5′-TCG CTA GTT GGC ATC GTT TAT G-3′, reverse: 5′-CGG AGG TTC GAA GAC GAT CA-3′). All samples were normalized using the 2^-ddCt^ method, with *18S rRNA* as the housekeeping gene.

### Cardiac morphology and ventricular contractility

*Tg (myl7:EGFP)* zebrafish larvae were anesthetized, and the beating heart was observed under a DMI6000B inverted fluorescence microscope (Leica Microsystems, Wetzlar, Germany). The heart of each individual zebrafish larva was imaged for 30 s and analyzed. Cardiac contractility was estimated as ventricular fractional shortening (vFS) by calculating the ventricular dimension (VD) at end-systole (VD_s_) and end-diastole (VD_d_). The formula was as follows: vFS = (VD_d_ – VD_s_) / VD_d_ × 100

### Blood flow and cardiac contraction irregularity

After the zebrafish larvae were anesthetized, blood flow data were obtained by real-time recording of blood flow in the dorsal aorta close to the heart for 30 s using the MicroZebraLab system (ViewPoint, Civrieux, France). Cardiac contraction irregularity was estimated as the standard deviation (SD) of the beat-to-beat interval, calculated based on blood flow data using MATLAB (MathWorks, Natick, MA, USA).

### Locomotion and survival analysis

The locomotion of zebrafish was analyzed using DanioVision and EthoVision XT (Noldus, Wageningen, Netherlands). Zebrafish larvae were individually placed in square 96-well plates with 200 μL of the egg water as a medium, and their movements were tracked and recorded for 5 min using DanioVision. During locomotion monitoring, motion was induced by stimulation with a tapping device every 30 s. Zebrafish locomotion was estimated by movement distance and duration, analyzed by EthoVision XT.

Kaplan–Meier survival analysis was used for survival analysis. Zebrafish larvae were transferred to a 96-well plate, one per well. Survival was observed using a microscope every 12 h until 9.5 dpf.

### Molecular docking analysis

A protein structure prediction model for zebrafish NHE1 was prepared using the AlphaFold Protein Structure Database, developed by DeepMind (London, UK) and EMBL-EBI (Cambridgeshire, UK)^27^. The 3D structures of GLU, EMPA, SOTA, and cariporide (CARI) for molecular docking analysis were obtained from PubChem. AutoDock Vina was used to analyze ligand binding sites and binding energy in zebrafish NHE1^28^.

### Drug affinity responsive target stability (DARTS)

The DARTS assay is an experimental method for identifying and studying protein‒ligand interactions^29^. We performed a DARTS assay to experimentally demonstrate the binding of SGLT inhibitors to zebrafish NHE1. More than 500 zebrafish larvae were euthanized and reacted with 0.2% trypsin EDTA and collagenase to separate them into single cells^30^. The protein was extracted by gently lysing the cells using M-PER (Thermo Fisher Scientific, Waltham, MA, USA). DMSO, EMPA, SOTA or CARI (Sigma‒Aldrich, St. Louis, MO, USA) was reacted with the prepared proteins at room temperature (RT) for 1 h. The reacted proteins were treated with pronase (Sigma‒Aldrich, St. Louis, MO, USA) for 30 min to induce proteolysis. The amount of each protein was confirmed by immunoblotting using antibodies against NHE1 (Thermo Fisher Scientific, Waltham, MA, USA) and glyceraldehyde-3-phosphate dehydrogenase (GAPDH, Abcam, Cambridge, UK).

### Measurement of intracellular H^+^, Na^+^ and Ca^2+^ concentrations

To measure intracellular H^+^, Na^+^, and Ca^2+^ concentrations, we used H9C2 cardiomyocytes cultured with 10% fetal bovine serum (FBS) in Dulbecco’s modified Eagle’s medium (DMEM, GenDEPOT, Katy, TX, USA) in black 96-well plates (Corning, New York, USA). To apply high glucose (HG) stimulation, cultured H9C2 cells were incubated with or without the addition of 40 mM D-glucose solution for 24 h after replacement of the medium with low-glucose DMEM (GenDEPOT, Katy, TX, USA). After treatment with EMPA, SOTA, or CARI for 2 or 24 h, cells were stained for intracellular ions. Staining for H^+^, Na^+^ or Ca^2+^ was performed using pHrodo Red AM (Thermo Fisher Scientific, Waltham, MA, USA), SBFI AM (Abcam, Cambridge, UK), or Fluo-4 AM (Thermo Fisher Scientific, Waltham, MA, USA), respectively, according to the manufacturer’s instructions. Staining was performed using a live cell imaging solution (LCIS, Thermo Fisher Scientific, Waltham, MA, USA) containing HEPES. After being washed with LCIS, the cultured cells were incubated at 37 °C for 30 min with pHrodo Red AM or Fluo-4 AM or at 37 °C for 60 min with SBFI AM. After the cells were stained with pHrodo Red AM, SBFI AM or Fluo-4 AM, fluorescence was measured using a SPARK multimode microplate reader (TECAN, Männedorf, Switzerland) at 560/580 nm, 340/500 nm, and 494/516 nm, respectively.

### Statistical analysis

All data are presented as the mean ± SD. Statistical analyses were performed with GraphPad Prism 8 (GraphPad Software, San Diego, CA, USA). Data were analyzed using Student’s t–test or the Mann–Whitney U–test to compare two groups or using one–way analysis of variance (ANOVA) followed by Tukey’s post hoc test to compare more than two groups. The Kaplan–Meier method with the log-rank test was used for survival analysis.

## Results

### Treatment with empagliflozin or sotagliflozin improved survival and locomotion in DM-HFrEF zebrafish

**Fig. 1:**
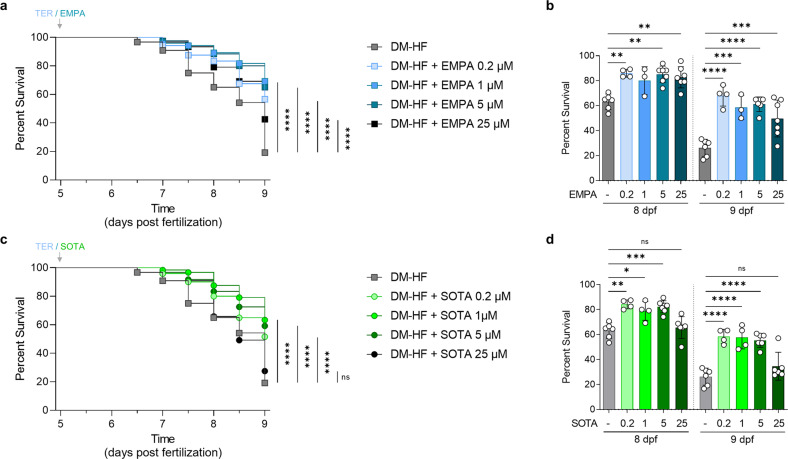
Comparison of survival rates in DM-HFrEF model zebrafish treated with empagliflozin or sotagliflozin. Survival analysis of DM-HFrEF zebrafish larvae after treatment with 0.2, 1, 5 and 25 μM empagliflozin or sotagliflozin. **a**, **c** Kaplan‒Meier survival curves (*n* = 120 larvae per group). **b**, **d** Ccross-sectional comparisons at the time points of 8 and 9 dpf and are presented as the mean ± standard deviation; each dot represents the value from one experiment (n = 3–6 experiments per group). The non-DM and DM groups were tested under the same conditions on the same day, and the graph is shown in Fig. 1 and Supplementary Fig. 2. *p < 0.05, **p < 0.01, ***p < 0.001, ****p < 0.0001 vs. the indicated group.

We first investigated the viability of DM-HFrEF zebrafish after treatment with various concentrations of EMPA or SOTA to determine whether these drugs increased the survival rate. The DM-HFrEF model had significantly reduced survival at 8 and 9 dpf (Supplementary Fig. 2), whereas the groups treated with 0.2, 1, and 5 μM EMPA or SOTA had significantly increased survival at 8 and 9 dpf (Fig. 1). Interestingly, a significant increase in survival was observed with 25 μM EMPA (Fig. 1a, b) but not with 25 μM SOTA (Fig. 1c, d).

**Fig. 2:**
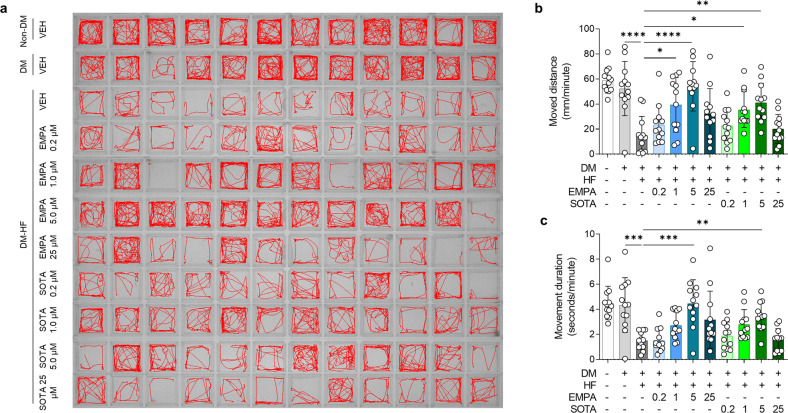
Comparison of locomotion in DM-HFrEF zebrafish treated with empagliflozin or sotagliflozin. **a** Representative locomotion tracking images of zebrafish larvae for a 5-minute period. **b**, **c** Average movement distance and movement duration per 1 min (*n* = 12 larvae per group). Data are presented as the mean ± standard deviation, and each dot represents the value for one zebrafish larva. *p < 0.05, **p < 0.01, ***p < 0.001, ****p < 0.0001 vs. the indicated group.

Next, we evaluated the changes in locomotion after treatment with various concentrations of EMPA or SOTA. In the DM-HFrEF zebrafish model, locomotion was further reduced after 24 h of TER treatment compared to the locomotion of the non-DM and DM-only groups. EMPA or SOTA treatment significantly preserved locomotion (Fig. 2a). In particular, 1 and 5 μM EMPA or SOTA significantly improved movement distance, whereas 0.2 and 25 μM EMPA or SOTA did not (Fig. 2b). Movement duration showed a similar trend. A significant increase was observed in the treatment groups given 5 μM EMPA or SOTA, whereas no significant difference was observed in the treatment groups given 0.2, 1 and 25 μM EMPA or SOTA (Fig. 2c).

**Fig. 3:**
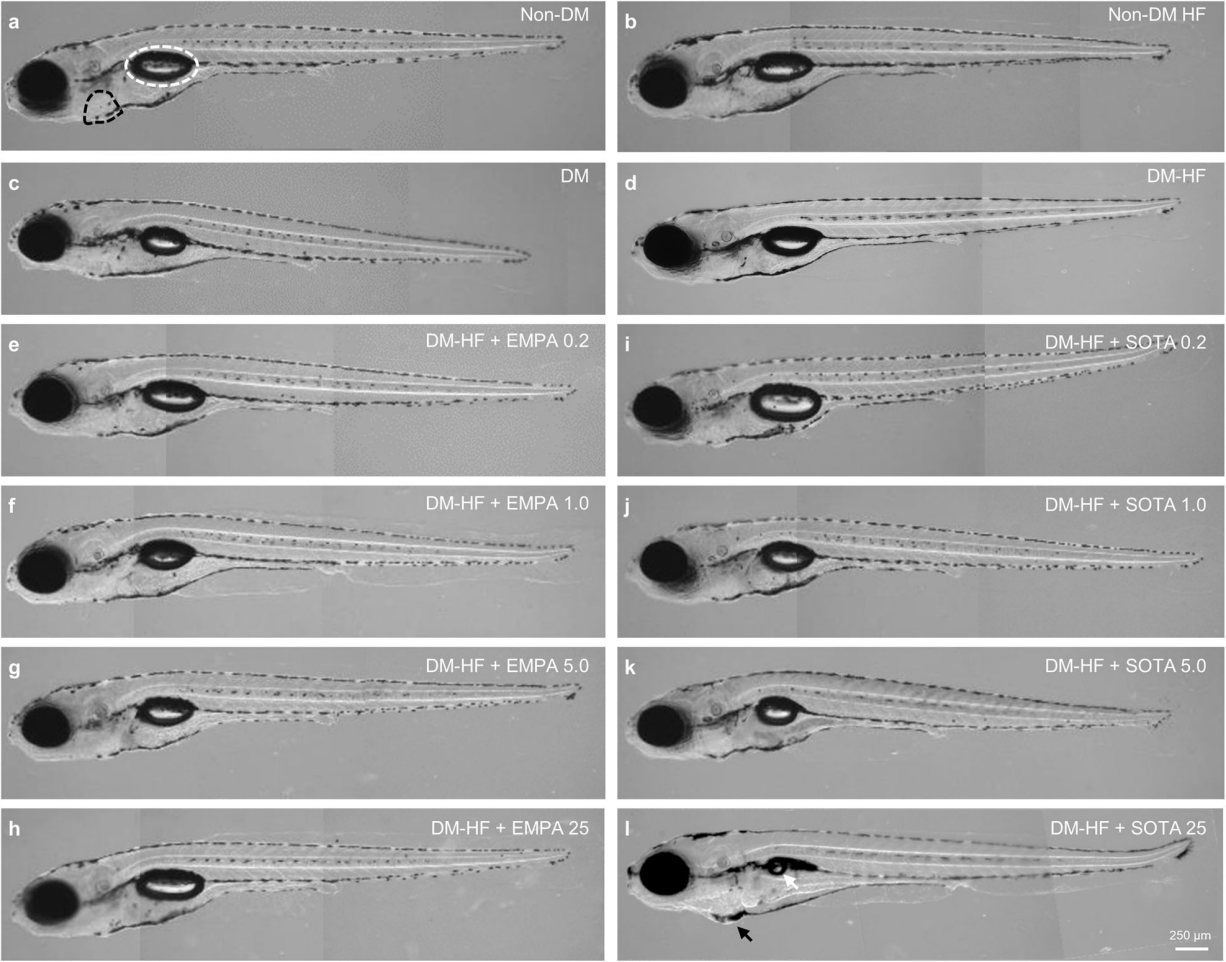
Comparison of morphology in DM-HFrEF zebrafish treated with empagliflozin or sotagliflozin. Representative morphological images. **a** Non-DM, **b** non-DM with HFrEF, **c** DM, **d** DM-HFrEF. **e**–**l** DM-HFrEF zebrafish following treatment with **e** 0.2 μM, **f** 1 μM, **g** 5 μM and **h** 25 μM empagliflozin or **i** 0.2 μM, **j** 1 μM, **k** 5 μM, and **l** 25 μM sotagliflozin. **a** The pericardium is indicated by a black dashed line, the swim bladder by a white dashed line, **l** the pericardial edema by a black arrow, and uninflated swim bladder by a white arrow.

When we evaluated the gross morphology of the zebrafish, we observed no significant difference in morphology between the groups, regardless of DM or HF conditions (Fig. 3a–d). In addition, morphological abnormalities were not observed in DM-HFrEF zebrafish treated with 0.2, 1, 5 and 25 μM EMPA or 0.2, 1 and 5 μM SOTA (Fig. 3e–k). Pericardial edema was observed in zebrafish larvae treated with 25 μM SOTA, and interestingly, a marked uninflated swim bladder was observed in these larvae compared to those in other groups (Fig. 3l and Supplementary Fig. 3). An uninflated swim bladder caused by high-molarity SOTA was observed not only in the DM-HFrEF zebrafish model but also in the non-DM zebrafish model of HF (Supplementary Fig. 4a, b).

### Treatment with empagliflozin or sotagliflozin improved cardiac function in a DM-HFrEF zebrafish model

Next, we compared the cardioprotective effects of EMPA with those of SOTA. Either EMPA or SOTA treatment preserved cardiac contractile functions in the DM-HFrEF zebrafish model, in which a marked decrease in cardiac functions was observed compared to non-DM or DM-only models (Supplementary Fig. 5).

**Fig. 4:**
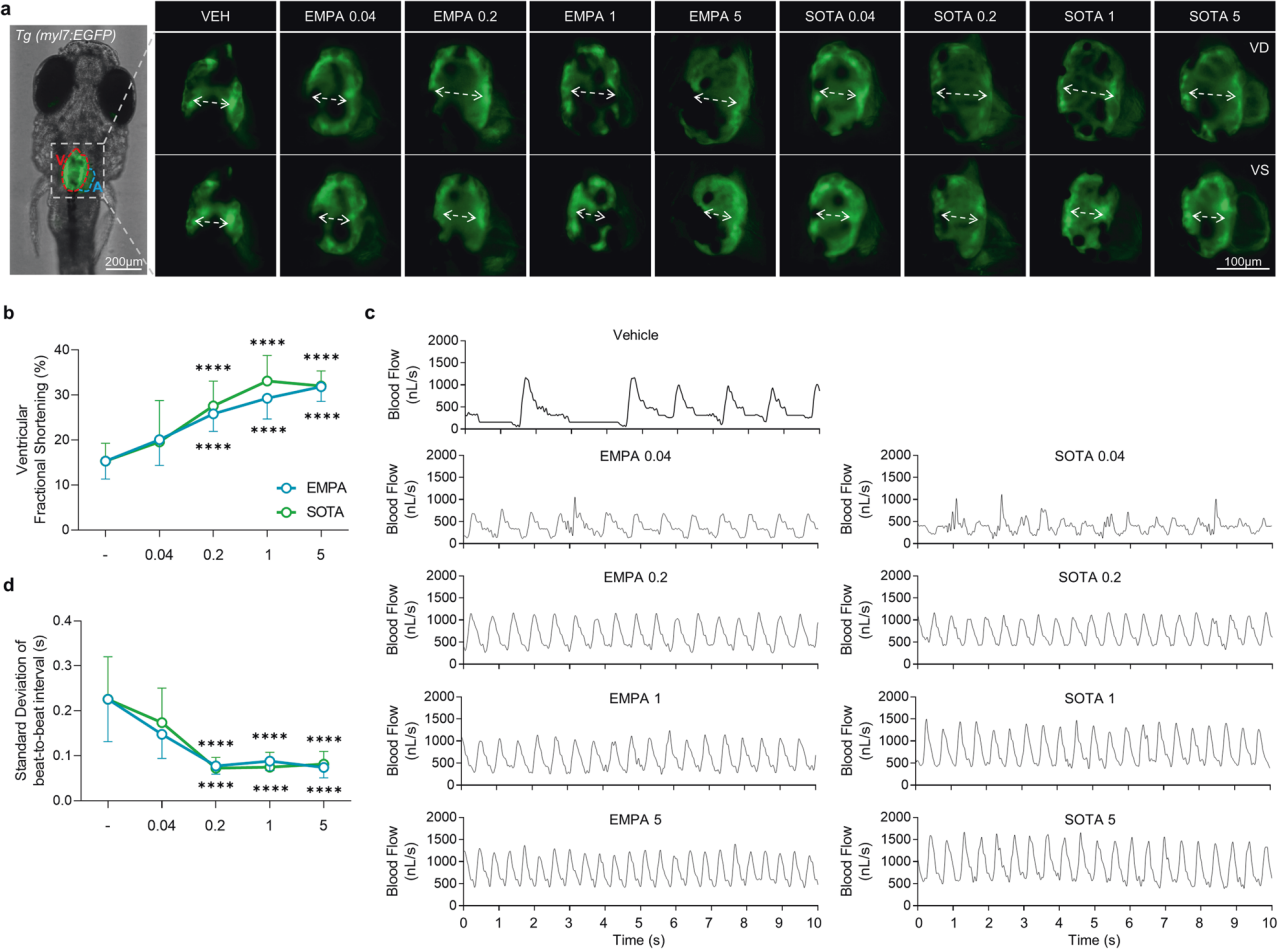
Comparison of cardiac contraction in DM-HFrEF zebrafish treated with various concentrations of empagliflozin or sotagliflozin. **a** Representative fluorescence microscopy images of *TG* (*myl7:EGFP*) zebrafish hearts at end-diastole and end-systole of ventricle. **b** Ventricular fractional shortening calculated based on fluorescent images (*n* = 8–25 larvae per group). **c** Representative blood flow graphs. **d** Standard deviation of the beat-to-beat interval analyzed based on blood flow (*n* = 14–25 larvae per group). **b**, **d** Data are presented as the mean ± standard deviation. ****p < 0.0001 vs. control group.

Ventricular contractility is estimated by the vFS parameter. There was no difference in vFS between non-DM and DM-only zebrafish, but a significant decrease in vFS was observed in DM-HFrEF zebrafish compared to DM-only zebrafish (Supplementary Fig. 5a, b). Treatment with various concentrations of EMPA or SOTA significantly enhanced the vFS of DM-HFrEF zebrafish. Treatment with 0.2–5 μM of both drugs significantly improved vFS, although no significant change was observed at 0.04 μM (Fig. 4a, b and Supplementary Movie 1). Notably, the vFS-preserving effect of EMPA peaked at 5 μM, whereas that of SOTA peaked at 1 μM (Fig. 4b). There was also no significant difference between groups in terms of heart morphology (Fig. 4a and Supplementary Movie 1).

The heart is a regular and constantly beating organ, and irregular contraction is a hallmark of HF. The SD of the beat-to-beat interval was calculated to quantify irregular contractions. More irregular contractions were observed in DM-HFrEF zebrafish than in the non-DM or DM-only group (Supplementary Fig. 5c). The most pronounced increase in the SD of the beat-to-beat intervals was observed in the DM-HFrEF zebrafish (Supplementary Fig. 5d). Treatment with various concentrations of EMPA or SOTA significantly suppressed irregular contractions in DM-HFrEF zebrafish (Fig. 4c). The SD of the beat-to-beat intervals in the DM-HFrEF zebrafish treated with 0.2–5 μM EMPA or SOTA was significantly preserved, but the same was not true with 0.04 μM EMPA or SOTA treatment (Fig. 4d).

In addition, *nppb*, a gene encoding the zebrafish form of the HF biomarker B-type natriuretic peptide, was markedly increased in DM-HFrEF zebrafish, whereas it was significantly decreased in both the EMPA- and SOTA-treated groups (Supplementary Fig. 6a). EMPA and SOTA treatments did not affect the expression of *ins*, a preproinsulin gene, and *pck1*, a phosphoenolpyruvate carboxykinase (PEPCK) 1 gene involved in gluconeogenesis, in contrast to the improvements in survival, locomotion, and cardiac function (Supplementary Fig. 6b, c).

### Both empagliflozin and sotagliflozin bound structurally to zebrafish NHE1 and inhibited its function

**Fig. 5:**
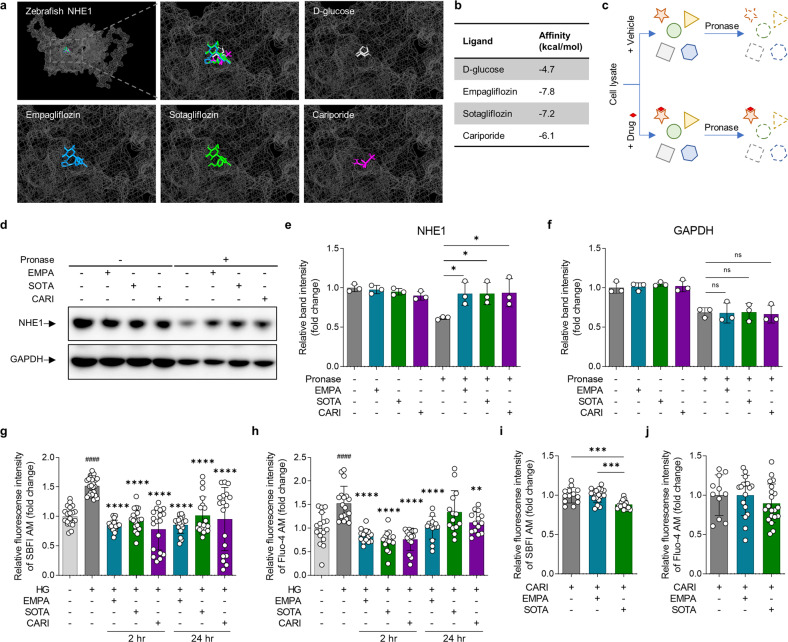
Comparison of structural binding and functional inhibitory effects of empagliflozin and sotagliflozin on NHE1. **a**, **b** Molecular docking analysis of empagliflozin, sotagliflozin, cariporide and D-glucose binding to a structural model of zebrafish NHE1 in silico. Empagliflozin is shown in blue, sotagliflozin is shown in green, cariporide is in purple, and D-glucose is in gray. D-glucose was used as a negative control, and cariporide was used as a positive control. **a** Binding site and **b** affinities. **c** Schematic illustration of the drug affinity responsive target stability (DARTS) assay in vitro. **d**–**f** DARTS analysis by immunoblotting with NHE1 and GAPDH antibodies (*n* = 3 samples per group). **g**–**j** Measurement of intracellular Na^+^ and Ca^2+^ to analyze the functional inhibitory effects of empagliflozin and sotagliflozin on NHE1. **g** Changes in intracellular Na^+^ and **h** Ca^2+^ concentrations in cardiomyocyte under HG condition were evaluated after treatment with (*n* = 14–27 samples per group). **i, j** Changes in intracellular Na^+^ and Ca^2+^ concentrations in response to empagliflozin or sotagliflozin treatment in cells pretreated with cariporide (*n* = 11–20 samples per group). Data are presented as the mean ± standard deviation, and each dot represents the value of each sample. **e**, **i** *p < 0.05, ***p < 0.001 vs. the indicated group. **g**, **h** ^####^*p* < 0.0001 vs. the LG group, **p < 0.01, ****p < 0.0001 vs. the HG group.

We performed in silico analysis of EMPA and SOTA binding to the predicted structural model of zebrafish NHE1. EMPA, SOTA, and the selective NHE1 inhibitor CARI were bound to the same site in zebrafish NHE1 (Fig. 5a and Supplementary Fig. 7). In addition, the binding affinities of EMPA, SOTA, and CARI were measured at −7.8, −7.2, and −6.1 kcal/mol, respectively; all three had higher binding affinities than the negative control, GLU (−4.7 kcal/mol, Fig. 5b).

We then compared the binding of EMPA and SOTA to zebrafish NHE1 using a DARTS assay in vitro (Fig. 5c). Pronase treatment resulted in the rapid proteolytic degradation of NHE1 in DMSO (VEH)-treated zebrafish protein, whereas proteolytic protection was observed in zebrafish protein reacted with EMPA, SOTA, or CARI (Fig. 5d, e). The NHE1 band intensity in the groups that were reacted with EMPA, SOTA, or CARI was significantly higher than that in the VEH group (Fig. 5e). However, GAPDH, which was used as a loading control, was consistently proteolyzed by pronase regardless of treatment with EMPA, SOTA, or CARI (Fig. 5d, f).

Finally, we confirmed the functional inhibition of NHE1 by EMPA and SOTA in vitro. The activation of NHE1 exchanges intracellular H^+^ with extracellular Na^+^, and the influx of Na^+^ is again exchanged with Ca^2+^ through the sodium-calcium exchanger (NCX), resulting in an increase in intracellular Na^+^, and Ca^2+^^31^. We evaluated whether these ion concentrations were changed by EMPA or SOTA in cardiomyocytes under high glucose (HG) conditions. Under HG conditions, intracellular Na^+^ and Ca^2+^ were increased compared to low glucose (LG) conditions, but treatment with EMPA, SOTA or CARI suppressed the changes in the intracellular Na^+^ and Ca^2+^ concentrations caused by HG (Fig. 5g, h). Similarly, treatment with EMPA, SOTA or CARI suppressed the slight concentration change in intracellular H^+^ mediated by HG, but there was no significant difference (Supplementary Fig. 8). These reductions were observed at both 2 h and 24 h after EMPA, SOTA or CARI treatment (Fig. 5g, h). The inhibitory effect on changes in intracellular Na^+^ and Ca^2+^ by EMPA or SOTA treatment showed a concentration-dependent tendency, and statistical significance was observed at 5 μM (Supplementary Fig. 9a, c, d). In particular, the intracellular Na^+^ concentration of cells treated with SOTA showed a significant difference from 0.04 to 5 μM SOTA (Supplementary Fig. 9b). After CARI pre-treatment, cardiomyocytes were treated with EMPA or SOTA, and intracellular Na^+^ and Ca^2+^ concentrations were measured. As a result, significant differences in intracellular Na^+^ and Ca^2+^ were not observed between the CARI-only group and the EMPA post-treatment group. In the SOTA post-treatment group, no significant results were observed for intracellular Ca^2+^, but the intracellular Na^+^ concentration decreased significantly in that group compared to the other two groups (Fig. 5i, j).

## Discussion

This study provides new insights into the protective effects of EMPA, a highly selective SGLT2 inhibitor, and SOTA, a dual SGLT1/2 inhibitor, against DM-HFrEF. First, at the same molarity, EMPA and SOTA exerted similar contraction regularity-improving, locomotion-preserving, and survival-promoting effects; EMPA was slightly superior to SOTA overall, but SOTA was superior to EMPA in preserving cardiac contractility. Moreover, the expected significant additive cardioprotective effect of SOTA was not observed in the DM-HFrEF zebrafish model. Second, the morphological abnormality and sharp decrease in survival rate observed in the high-dose SOTA-treated group imply the possibility of side effects of SOTA in zebrafish larvae. Third, both EMPA and SOTA inhibited NHE1 structurally and functionally, which may be the main mechanism underlying their cardioprotective effect.

Newly developed diabetes drugs, including SGLT2 and dual SGLT1/2 inhibitors, provide an effective blood-glucose-lowering effect^11,14^. These inhibitors have an excellent cardioprotective effect in addition to their use as diabetes drugs. According to the EMPA-REG and SOLOIST trials, both drugs dramatically reduced hospitalization for HF and overall mortality in patients with DM^8,10^. In addition, in experiments using an animal model of DM, treatment with EMPA or SOTA preserved cardiac function by inhibiting myocardial fibrosis, hypertrophy, and inflammation^32,33^. However, studies comparing the cardioprotective effects of EMPA and SOTA are still insufficient. We compared the beneficial effects of these two drugs for the first time, focusing on their cardioprotective effects. The results of this study using the DM-HFrEF zebrafish model show that treatment with each of these drugs has a remarkable and similar cardioprotective effect and survival-promoting effect.

SOTA is the first dual SGLT1/2 inhibitor with high selectivity for both SGLT1 and SGLT2^14^. SGLT1 is expressed in the myocardium^34^. SGLT1 in cardiomyocytes contributes to glucose uptake and plays an essential role in pathological heart conditions^35^. SGLT1 inhibition helps decrease myocardial hypertrophy and fibrosis^36^. As such, SOTA is expected to provide a cardioprotective effect by the same mechanism as SGLT2 inhibitors, in addition to a cardioprotective effect through SGLT1 inhibition, therefore offering a higher cardioprotective effect than single-specificity SGLT2 inhibitors. However, in our study, the two inhibitors conferred similar cardioprotective effects and survival rate improvements at various molarities (0.2–5 μM). The maximal cardiac effects of the two drugs were similar. A slight difference between the two drugs was observed in vFS, one of the variables used herein to evaluate cardiac function. SOTA reached its maximum effect at a lower concentration than EMPA, but the difference was not significant. Although SOTA inhibits SGLT1, the similarly confirmed cardioprotective effects of the two drugs suggest that NHE1 inhibition rather than SGLT1 inhibition is the main mechanism behind this effect in the DM-HFrEF zebrafish model. However, further studies are needed to analyze the contribution of NHE1 and SGLT1 inhibition to cardiac function protection. In clinical practice, the doses of these drugs are 10 and 25 mg EMPA or 200 mg SOTA based on clinical trials for DM patients^37,38^. Despite the clinical use of much higher doses of SOTA than EMPA, our study results show that both drugs provide a significant cardioprotective effect even at low molarities. In addition, high-molarity SOTA significantly decreased the survival rate compared to the same molarity of EMPA, and pericardial edema and an uninflated swim bladder were observed. These results suggest that treatment with a high molarity of SOTA may have side effects in zebrafish. Furthermore, although the swim bladder in zebrafish is evolutionarily homologous to the mammalian lung^39^, it is unclear whether SOTA-induced swim bladder abnormalities in zebrafish predict corresponding lung toxicity in mammals.

As SGLT2 is not expressed in cardiomyocytes, several studies focusing on the mechanism of the cardioprotective effect of SGLT2 inhibitors have focused on NHE1 as an off-target ligand of SGLT2 inhibitors^15,16^. Induction of NHE1 expression and activation is increased by DM-related stimuli^40^. Upregulation of NHE1 is found not only in patients with DM but also in the ventricular tissue of patients with HF^18^. In addition, selective inhibition of NHE1 improved cardiac function by inhibiting fibrosis and cardiac hypertrophy in an experimental HF model^19^. These reports suggest that NHE1 is a molecule that plays an essential role in the pathogenesis of DM-HFrEF and has potential as a novel target of therapeutic strategies for DM-HFrEF; however, this mechanism remains controversial^16,17^. We showed that both EMPA and SOTA structurally bind to NHE1 and inhibit its functions both in silico and in vitro. The possibility of binding was suggested by a molecular docking assay and a DARTS assay, and the possibility of inhibition was shown by measuring changes in intracellular Na^+^ and Ca^2+^. In particular, inhibitor competition assays of CARI and EMPA provided clear evidence that EMPA inhibits NHE1, and the results for CARI and SOTA may be related to other mechanisms involved in SGLT1 inhibition. These results not only corroborate several previous studies^15,16^ but also provide the first evidence that SOTA, a dual SGLT1/2 inhibitor, directly inhibits NHE1, just as single-specificity SGLT2 inhibitors including EMPA inhibit NHE1.

This study has several limitations. First, although the focus was on the inhibition of NHE1, to elucidate the exact molecular mechanism of the cardioprotective effect, it is necessary to study the interactions more precisely through loss-of-function and gain-of-function experiments on NHE1, SGLT1, and SGLT2. Second, although the use of zebrafish as an animal model in this study has various advantages, it may be difficult to apply the results to humans because zebrafish are nonmammalian. Third, the inhibitory effect of SGLT inhibitors on NHE1 was confirmed only in cardiomyocytes; it is still necessary to confirm the effects of SGLT inhibitors in various cells other cells constituting the heart, such as endothelial cells and immune cells. Finally, the cause of the decreased survival and morphological abnormalities observed in the high-dose SOTA-treated group has not yet been elucidated.

In conclusion, this study showed that EMPA, a highly selective SGLT2 inhibitor, and SOTA, a dual SGLT1/2 inhibitor, provide similar cardioprotective effects in a zebrafish model of DM-HFrEF. No significant differences in protective effects were observed due to the expected SGLT1 inhibition by dual SGLT1/2 inhibitors. However, both inhibitors showed a high binding affinity for NHE1. Therefore, we propose that NHE1 inhibition is an essential mechanism for the cardioprotective effects of SGLT2 and dual SGLT1/2 inhibitors. This study will help researchers understand the mechanisms by which SGLT2 inhibitors and dual SGLT1/2 inhibitors affect DM-HFrEF and provide important information about the potential benefits of these inhibitors for DM patients with HF.

### Supplementary information

Supplementary data

Supplementary Movie 1
