# Author Correction: Comparison of the effects of empagliflozin and sotagliflozin on a zebrafish model of diabetic heart failure with reduced ejection fraction

**DOI:** 10.1038/s12276-023-01154-2

**Published:** 2024-01-15

**Authors:** Inho Kim, Hyun-Jai Cho, Soo Lim, Seung Hyeok Seok, Hae-Young Lee

**Affiliations:** 1 Department of Internal Medicine, Seoul National University Hospital, Seoul, Korea; 2 Department of Microbiology and Immunology, Seoul National University College of Medicine, Seoul, Korea; 3 Department of Internal Medicine, Seoul National University College of Medicine, Seoul, Korea; 4 Department of Internal Medicine, Seoul National University Bundang Hospital, Seongnam, Korea; 5 Department of Biomedical Sciences, Seoul National University College of Medicine, Seoul, Korea; 6 Cancer Research Institute, Seoul National University, Seoul, Korea

**Keywords:** Heart failure, Homeostasis

Correction to: *Experimental & Molecular Medicine* 10.1038/s12276-023-01002-3, published online 01 June 2023

After the online publication of this article, the authors noticed an error in the Results section.

The correct statement of this article should have read as below.

In the original article, there was an error in Fig. 3d using a duplicate image of the same DMHF group as in a previous study conducted simultaneously with this study. There was no change in the morphology of DMHF in both the previous study and this study. However, this study explained the changes in morphology caused by the treatment of Empagliflozin and Sotagliflozin in the DMHF model based on previous research results. Therefore, the misuse of the image did not affect the results and scientific conclusions of the article. We would now like to replace Fig. 3d to provide a new, more representative Fig. 3 as follows:
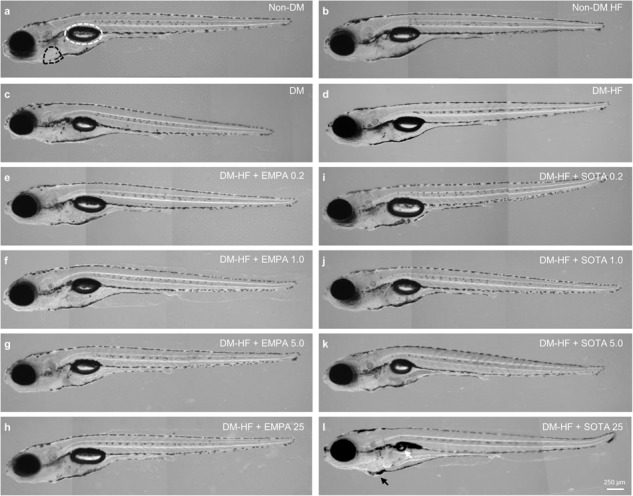


The authors apologize for any inconvenience caused.

The original article has been corrected.

