# Supplementary material for: Comparison of the effects of empagliflozin and sotagliflozin on a zebrafish model of diabetic heart failure with reduced ejection fraction
Source: Exp Mol Med. 2023 Jun 1;55(6):1174–81. doi: 10.1038/s12276-023-01002-3 (PMC10318005; doi:10.1038/s12276-023-01002-3)
Supplement: Supplementary file 1 — Supplementary data [file 12276_2023_1002_MOESM1_ESM.pdf]

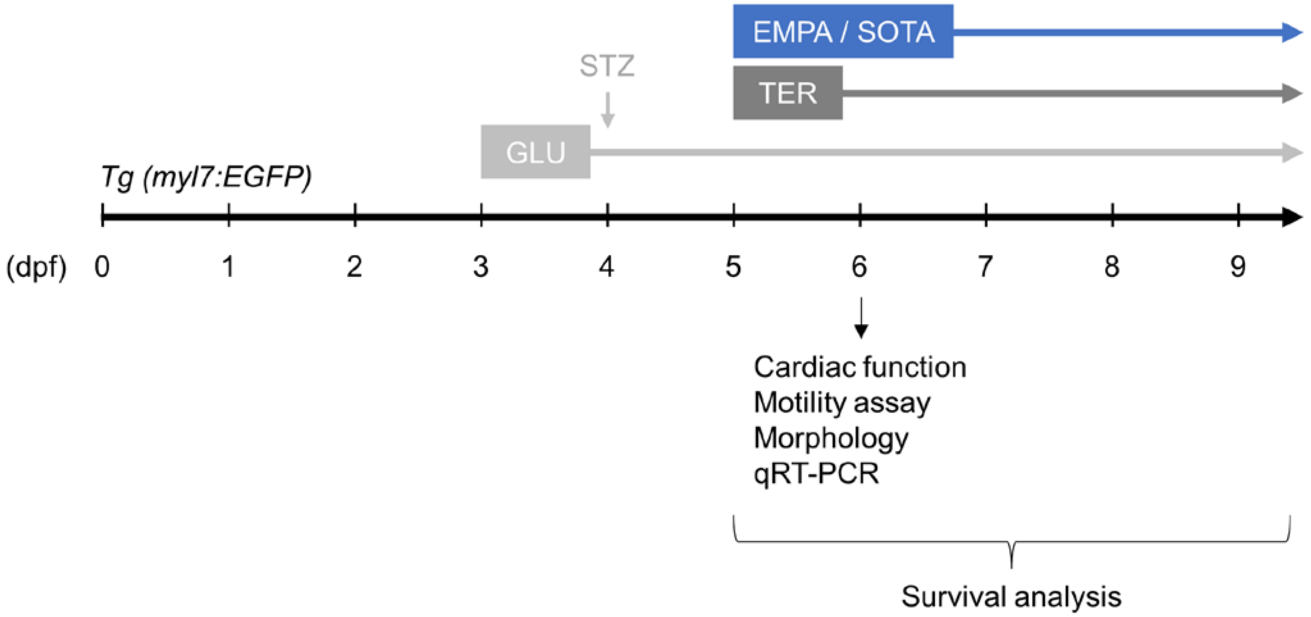

Supplementary Fig. 1. Schematic of the study design.

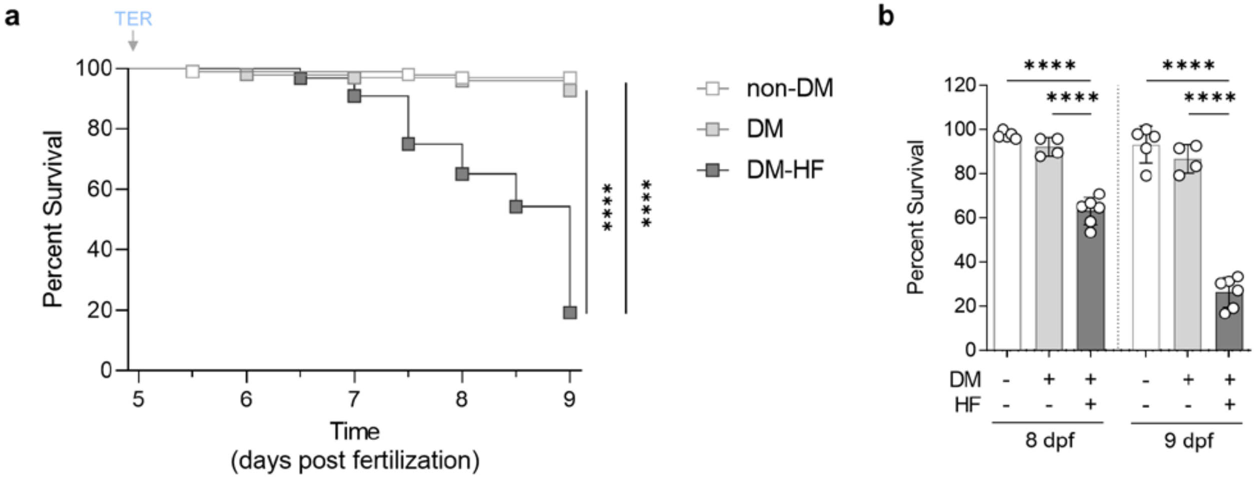

Supplementary Fig. 2. Survival rates of non-DM, DM and DM-HF zebrafish. (**a**) Kaplan-Meier survival analysis of non-DM, DM, and DM-HF zebrafish larvae. (**b**) Survival rate at 8 and 9 dpf (n = 96-120 per group). All groups were tested under the same conditions, and the graphs are shown to Fig. 1 and Supplementary Fig. 2. Data are presented as the mean ± standard deviation and each dot represents the value of each experiment. ****p<0.0001 vs. indicated group.

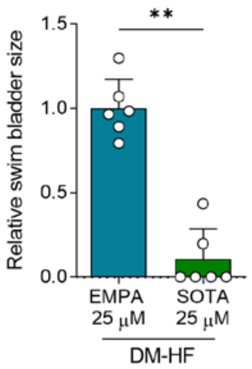

Supplementary Fig. 3. Defect of swim bladder in high molarity sotagliflozin treated DM-HFrEF zebrafish. Relative swim bladder size in DM-HF zebrafish treated with 25 μM empagliflozin or sotagliflozin (n = 6 per group). Data are presented as mean ± standard deviation and each dot represents the value of each zebrafish larva. ** *p* < 0.01 vs. indicated group.

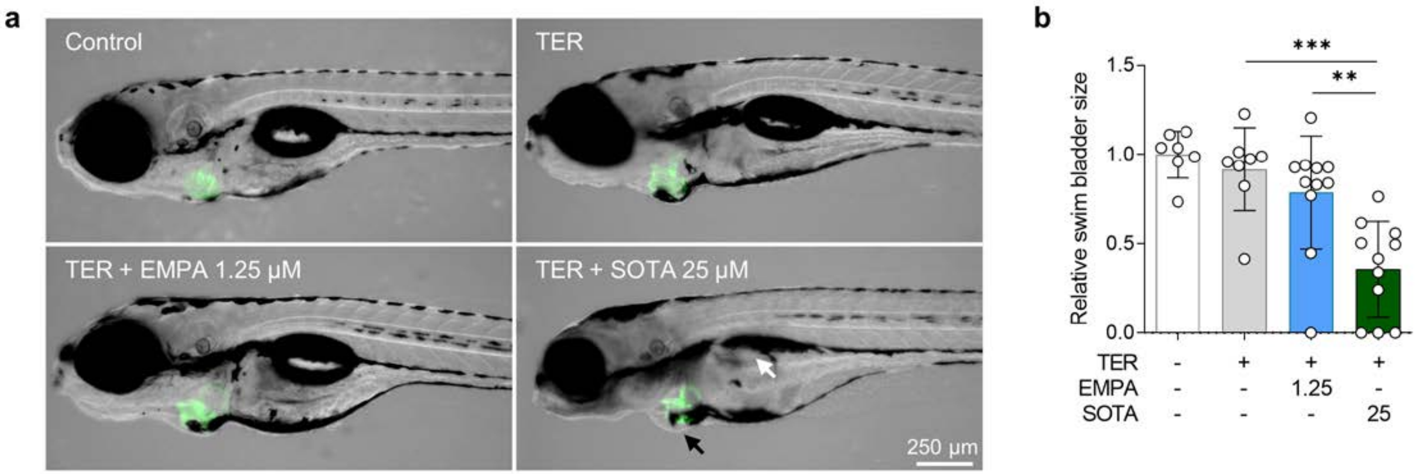

Supplementary Fig. 4. Pericardial edema and uninflated swim bladder in high dose sotagliflozin treated HF induced non-DM zebrafish. (**a**) Representative morphological images. The pericardial edema is indicated by a black arrow, and uninflated swim bladder is indicated by a white arrow. (**b**) Relative swim bladder size in non-DM with HF zebrafish treated with 1.25 μM empagliflozin or 25 μM sotagliflozin (n = 7-11 larvae per group). Data are presented as mean ± standard deviation and each dot represents the value of each zebrafish larva. ** $p < 0.01$, *** $p < 0.001$ vs. indicated group.

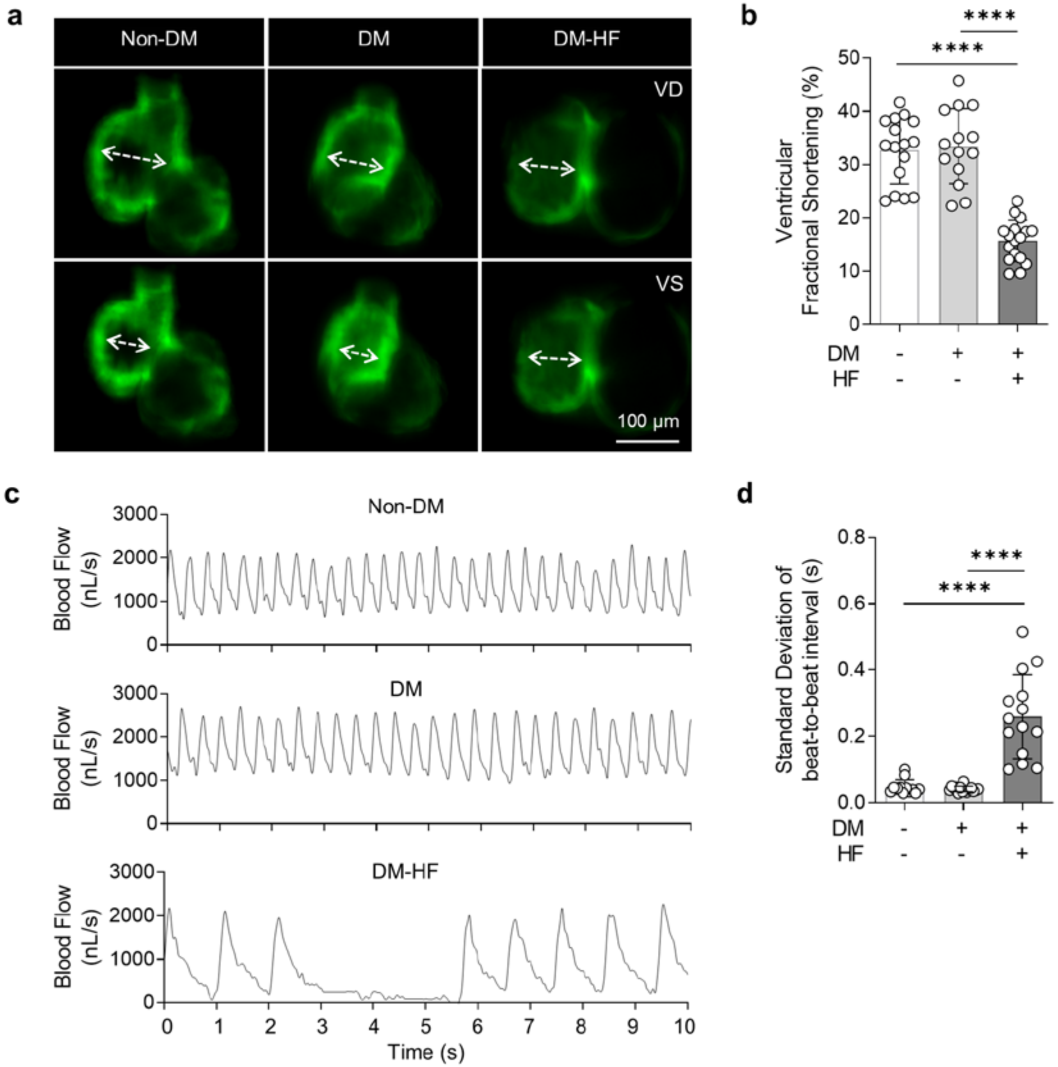

Supplementary Fig. 5. Cardiac contractile functions in the DM-HFrEF zebrafish model. (**a**) Representative fluorescent microscopy images of *Tg* (*myl7:EGFP*) zebrafish heart with a green fluorescent protein (GFP). (**b**) Ventricular fractional shortening calculated based on fluorescent images. (n=14-17 per group). (**c**) Representative blood flow graphs. (**d**) Standard deviation of beat-to-beat interval analyzed based on blood flow (n = 12-15 per group). Data are presented as mean ± standard deviation and each dot represents the value of each zebrafish larvae. ****p < 0.0001 vs. indicated group.

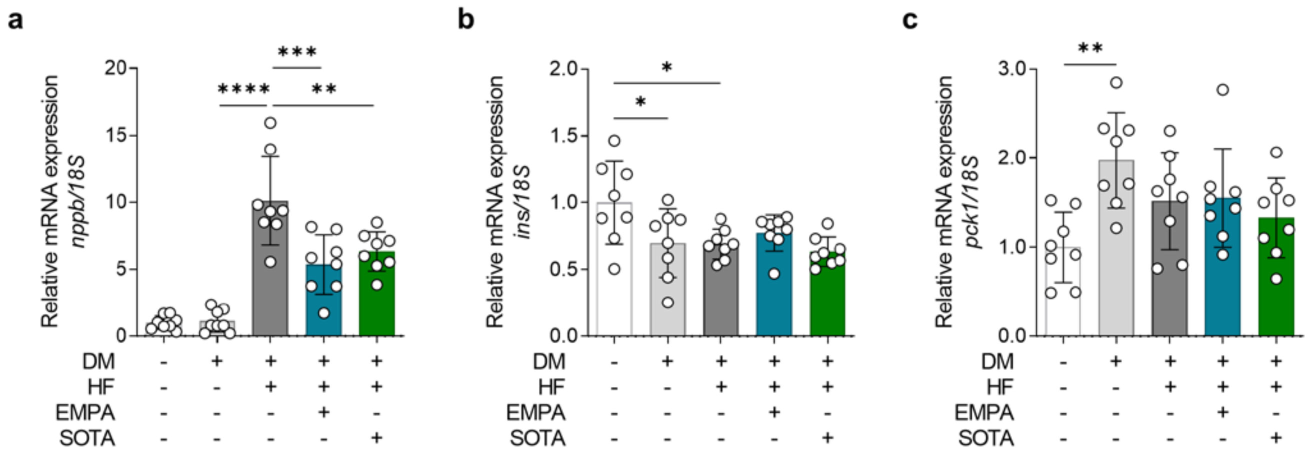

Supplementary Fig. 6. Expression of heart failure marker and glucose metabolism-related genes in DM-HF zebrafish treated with empagliflozin or sotagliflozin. (**a**) Relative mRNA expression of *nppb* (**b**) *ins* and (**c**) *pck1* (n = 8 per group). Each group had 8 samples, with 10 larvae per sample. Data are presented as mean ± standard deviation and each dot represents the value of each sample. * $p < 0.05$, ** $p < 0.01$, *** $p < 0.001$, **** $p < 0.0001$ vs. indicated group.

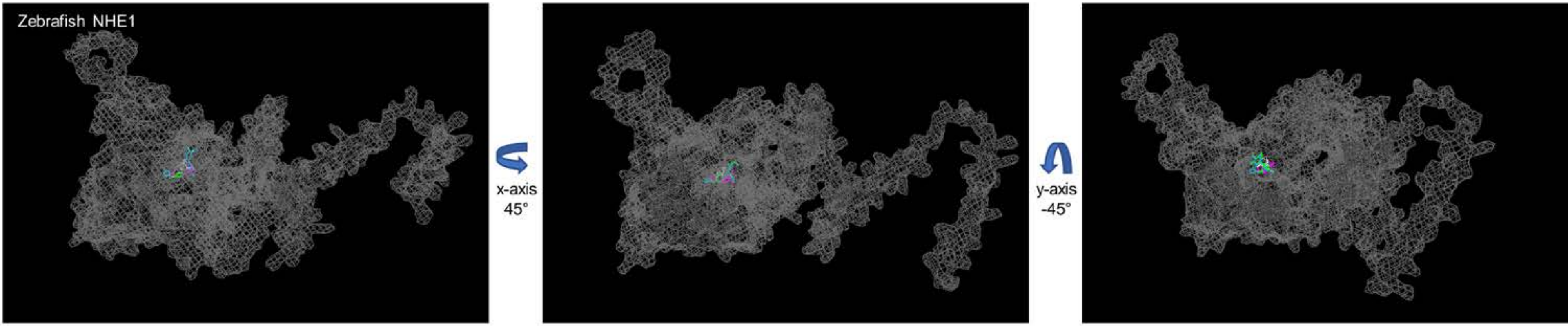

Supplementary Fig. 7. Orientation of the zebrafish NHE1 structural model for molecular docking analysis *in silico*. Orientation strategy of the zebrafish NHE1 structure prediction model for molecular docking analysis of empagliflozin, sotagliflozin, cariporide and D-glucose.

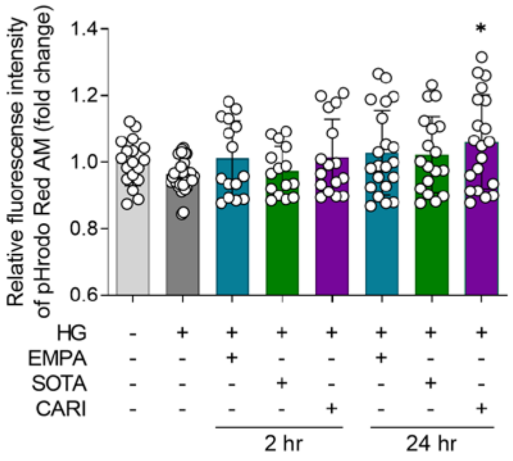

Supplementary Fig. 8. Intracellular H+ concentration in empagliflozin or sotagliflozin treated cells. pHrodo Red AM for intracellular H^+ (n = 15-34 samples per group). Data are presented as mean ± standard deviation and each dot represents the value of each sample. * p < 0.05, *** vs. HG group.

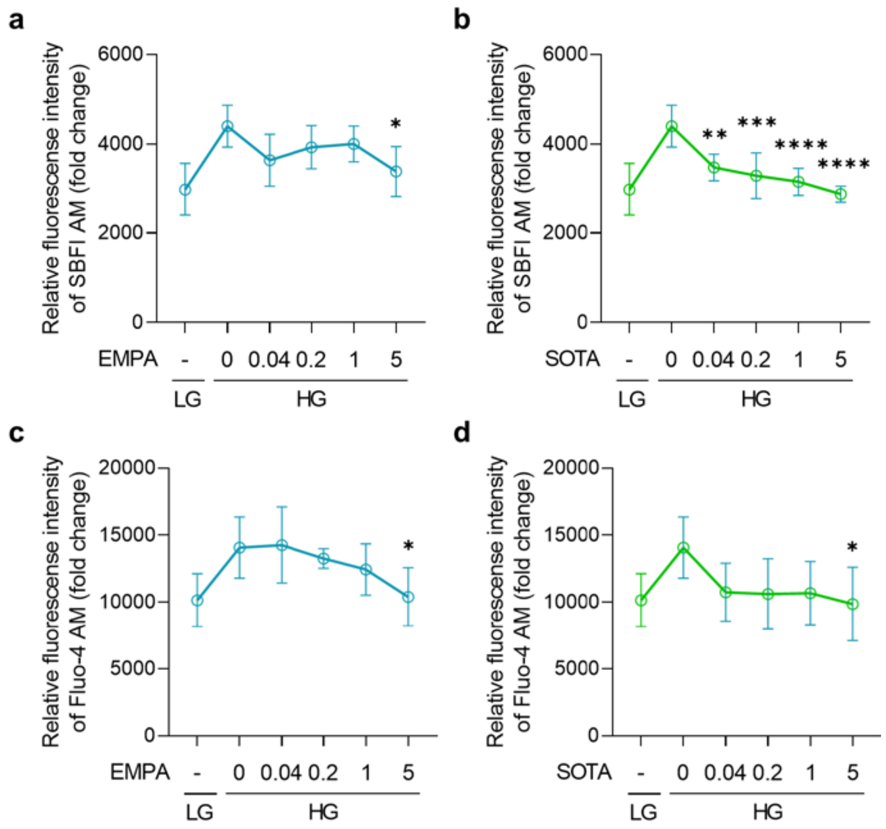

Supplementary Fig. 9. Intracellular Na^+ and Ca^{2+} concentrations treatment with empagliflozin or sotagliflozin in a dose-dependent manner. Measurement of intracellular Na^+, and Ca^{2+} for the analysis of the NHE1 functional inhibitory effect of empagliflozin or sotagliflozin. (**a** and **b**) SBFI AM for intracellular Na^+, and (**c** and **d**) Fluo-4 AM for intracellular Ca^{2+} (n = 6-8 samples per group). Data are presented as mean ± standard deviation and each dot represents the value of each sample. * $p < 0.05$, ** $p < 0.01$, *** $p < 0.001$, **** $p < 0.0001$ vs. HG group.

	Empagliflozin	Sotagliflozin
Molarity (μM)	Concentration (mg/L)	
0.2	0.0902	0.0850
1	0.4509	0.4249
5	2.2546	2.1247
25	11.2728	10.6235

Supplementary Table 1. Molarity of Empagliflozin and Sotagliflozin used in the experiment
